# A qualitative case study of body image in women with breast cancer participating in an exercise program

**DOI:** 10.1007/s00520-025-09963-z

**Published:** 2025-10-01

**Authors:** Pau Mateu, Clara Teixidor-Batlle, María-Pilar Suárez-Alcázar, Pablo Salas-Medina, Ignacio Catalá-Vilaplana, Carlos Hernando-Domingo, María Muriach, Eladio Collado-Boira

**Affiliations:** 1 Institut Nacional d’Educació Física de Catalunya (INEFC Pirineus), Universitat de Lleida, Lleida, Spain; 2 University of Vic - Central University of Catalonia, Sport, Exercise and Human Movement Research Group (SEaHM), Vic, Spain; 3 Grup d’Investigació Social I Educativa de L’Activitat Física I L’Esport (GISEAFE), Barcelona, Spain; 4 Nursing Department, University of Jaime I, Castellón de La Plana, Spain; 5 Department of Physical Therapy, Faculty of Medicine, University of British Columbia, Vancouver, Canada; 6 Department of Education and Specific Didactics, University of Jaume I, Castellón de La Plana, Spain; 7 Medicine Department, University of Jaime I, Castellón de La Plana, Spain

**Keywords:** Psycho-Oncology, Cancer recovery, Physical exercise intervention, Self-concept

## Abstract

**Purpose:**

To analyze the body image perceptions of female breast cancer patients who took part in a physical exercise (PE) program.

**Methods:**

Four discussion groups were held with 40 breast cancer patients participating in an exercise program. Hierarchical content analysis identified three central themes: (a) consequences of cancer, (b) coping, and (c) consequences of exercise.

**Results:**

Regarding cancer consequences, narratives linked to the others’ perception and body stigma prevailed. Participants used mutual support and empowerment as their main coping strategies. The PE program was perceived as a promoter of physical (functional and aesthetic) and psychological benefits.

**Conclusions:**

The participation of female breast cancer patients in PE programs contributes to greater physical, psychological, and social well-being. The analyzed PE program contributed, in general, to an improvement in body image with special emphasis on body function and in cognitive and affective dimensions.

Implications for cancer survivors.

PE programs can be a valuable intervention for breast cancer survivors, enhancing body image, quality of life, and emotional resilience during recovery.

## Introduction

Breast cancer is one of the most prevalent diseases among women. It accounts for a quarter of all cancers in women and is the most commonly diagnosed cancer and the leading cause of cancer death in this population [1]. In 2020, an estimated number of 685,000 women died due to this disease, accounting for 16% of all cancer deaths in women [2]. However, breast cancer has high survival rates in northern countries such as Spain (over 85% survival within 5 years of diagnosis) [3].

The most common and effective treatments against breast cancer combine surgical procedures with complementary local therapies (e.g., radiotherapy) or systemic treatments (e.g. chemotherapy, hormonal therapy) [4]. Despite the high survival rate of female breast cancer patients in the northern countries, the treatments used are associated with side effects that significantly reduce the quality of life of these patients and this must, therefore, be considered [5]. On the one hand, surgeries can generate problems such as pain and loss of mobility in the surgical area, loss of muscle mass resulting from immobility, and/or lymphedema caused by lymph nodes resection [6]. On the other hand, systemic treatments can lead to problems such as osteoporosis or joint pain [7]. Some of these side effects are directly related to physical appearance, and can contribute to the development of a negative body image [8]. In this sense, side effects can lead to unfavorable perceptions and predominantly negative thoughts and feelings in relation to the body, as well as maladaptive actions or health-risk behaviors [9].

In this context, it is noteworthy that physical exercise not only significantly enhances the quality of life during and after cancer treatment but also has the potential to reduce the risk of developing certain types of cancer, decrease cancer-specific mortality by nearly 20%, and alleviate various side effects associated with oncological treatments [10]. Additionally, physical exercise has a profound impact on the psychological and emotional well-being of cancer patients, fostering social interaction, and enhancing perceptions of self-efficacy and functionality, which may, in turn, improve body image and self-esteem [11]. Furthermore, our research group has recently demonstrated that PE programs, as an adjunctive intervention, are effective not only in preventing the recurrence of treatment-related side effects in breast cancer patients but also in promoting mental well-being [12]. However, such interventions are not currently covered by public health services. Therefore, it is imperative to advance research in this area to generate robust evidence that supports the inclusion of PE programs as an additional adjunctive therapy within public health systems. In line with this objective, the aim of the present study was to assess the body image perceptions of female breast cancer patients who participated in a PE program.

## Methods

### Study design

This research was conceived from a qualitative perspective, as there was a need to know in depth the perceptions of female breast cancer patients in a specific context [13]. Within this framework, the inquiry was conducted as an instrumental case study, as it is appropriate when analyzing particular cases to deepen the understanding of a broader issue and/or to refine theoretical generalizations. The study, which is descriptive and retrospective in nature, is positioned within the postpositivist paradigm. This philosophical framework is in line with critical realist ontology, since it assumes the existence of an objective reality external to the observer which, in turn, can only be imperfectly grasped, as observations are fallible. Consequently, any theory can be tested and reformulated. Moreover, postpositivism is associated with a modified objectivist epistemology in which researchers can approach external reality but are not separated from it. In this sense, researchers’ personal values and social positions can influence their understanding of the reality under study [14].

The present case study was carried out with women diagnosed with breast cancer (stages I–IV) actively undergoing treatment (chemotherapy, radiotherapy, hormone therapy) who enrolled in a PE program performed by the Chair of Physical Activity and Oncology «Fundación José Soriano Ramos» of the Universitat Jaume I (Spain). This organization, through different lines of work addresses the relationships between different types of cancer, physical activity and quality of life. The PE program for female breast cancer patients is one of the most prominent and recognized projects at a social level. This study’s approach allows for a holistic understanding of how our PE program influences the patient’s body image perceptions, considering contextual and individual variables that would be difficult to convey through other methodologies.

### Sampling and recruitment

In accordance with the Declaration of Helsinki and following the approval from the Jaume I University ethics committee (CEISH/75/2023), participant recruitment was conducted at the Medical Oncology Service of the Provincial Hospital Consortium of Castelló, according to the same inclusion criteria outlined in our previous study [12]. Potential participants were contacted to confirm their interest and to clarify the study’s objectives, as well as the relevant ethical considerations. They were also requested to read and sign an informed consent document prior to engaging in the focus group. Finally, a total of 40 women, aged between 34 and 62, volunteered to take part in the study. The intervention consisted of a 24-week personal training program guided and supervised by a graduate in physical activity and sports with specialization in exercise oncology. Breast cancer patients engage twice a week in 60-min sessions, and carry out different physical exercises (mainly strength and aerobic exercises) at the sport facilities of Jaume I University.

#### Data collection

We chose to hold discussion groups for data collection, since it offers the possibility for several people to collaboratively share ideas, feelings and perceptions around one or more topics. The focus groups were conducted face to face, in a semi-structured style of interviewing. We designed an interview guide based on open questions in order to encourage participation (see Appendix). This type of interview guide offers flexibility for researchers since they do not need to ask the same questions in all discussion groups, thus, facilitating a more fluid conversation because the participants have greater control. Also, its structure is strict enough to be able to collect important information about the object of study [15, 16].

Favoring a methodological naturalism [17], four focus groups included 10 participants of the same exercise groups. Bringing together women who were already part of the same exercise groups facilitated familiarity, reduced inhibitions, and encouraged open sharing during the discussions. All focus groups were conducted between June 1 st and June 30th, 2023, in a meeting room equipped with ambient microphones, at Jaume I University. Participants were arranged in a semi-circle format in order to facilitate interaction and ensuring that all voices were recorded during the discussions. At the beginning of each session, the purpose of the study and ethical considerations were reminded, and the anonymity of the participants and data confidentiality were guaranteed. The focus groups lasted an average of 65 min (range, 59–73 min). All of them were recorded and transcribed verbatim.

The interview guide initially consisted of an introductory part in which terminological issues such as the concept of body image itself were discussed. This was done through questions such as:

“What does body image mean to you?”

The introductory phase of conceptual clarification facilitated the further development of the discussion group on the basis of a common understanding of the expectations and central concepts. Also, it was useful to introduce participants to the dynamics of the activity (for many, it was the first time they participated in a study of these characteristics) and in preparation to address other more complex and personal issues. After the introductory part, participants were asked about their perception in body changes after being diagnosed with breast cancer, the role physical exercise PE played in terms of their body image perception, etc. At the end of the focus groups, participants were invited to offer recommendations and arguments for other female breast cancer patients who were considering to participate in a PE program similar to the one discussed in the focus group. This final part helped to reduce the tension after a potentially intense discussion.

#### Data analysis

The authors followed the Reporting Standards for Qualitative Research set by the American Psychological Association (2020) [18] and the Consolidated criteria for reporting qualitative studies (COREQ) [19]. Due to the design and approach of the research, discussion groups were considered as an appropriate data collection method to capture relevant information in order to respond to the purpose of the study. Furthermore, this work was carried out by a multidisciplinary team of researchers with diverse academic backgrounds, institutional roles, and fields of knowledge (sports science [sociocultural aspects of physical activity and sport, sport psychology, physical activity and health], medicine). The team included both women and men from different Spanish universities and with varying levels of academic and professional experience, ranging from early career researchers with doctoral degrees and doctoral candidates to university professors with many years of teaching and research experience. In terms of their relationship with the study participants, some authors were directly involved in the physical exercise programs and knew the women personally, while others had no previous contact with them. This combination of “insider” and “outsider” perspectives, together with the diversity of academic training, professional seniority, and gender within the team, shaped how the phenomenon under study was approached, questioned, and interpreted. This diversity contributed to investigator triangulation, as several rounds of discussion among the authors enriched the (re)design of the study and the (re)interpretation of the data. In addition, our findings are evidence-based since we provide relevant fragments of the stories written by participants.

To ensure the trustworthiness and credibility of the data analysis, a six-step, hierarchical, content analysis of inductive nature was conducted. This analysis allows to compare and contrast the data, to divide the data into broader or narrower categories, as well as to identify and organize the material collected. Therefore, it facilitates the development of general knowledge about a specific topic [16]. In an initial step of immersion, authors deeply examined the transcripts to acquaint themselves with the data. The second step consisted in inductively identifying and coding themes, focusing on text fragments interpreted as relevant to the research purpose. Thirdly, after completing the coding process, authors connected and ordered the established categories by clustering them around three overarching themes that seemed to be meaningful to the participants, namely:

- Cancer consequences,
- Coping, and
- Physical exercise consequences.

The fourth step of this process involved reviewing the original transcripts, comparing them with the thematic structure crafted from the authors’ interpretation, and discussing their coherence level. Additionally, it entailed assessing whether any pertinent information had been inadvertently omitted from the analysis. To further enhance credibility, in a fifth step, a critical peer with expertise in qualitative research reviewed the analytical process and agreed that the hierarchical content analysis provided a suitable and precise depiction of the body image perceptions of women diagnosed with breast cancer who took part in a PE program. This process aligns with the technique of peer debriefing. It is remarkable that, an audit trail was maintained throughout the analysis to ensure comprehensive documentation of the whole analytical process. Although member checking was not formally conducted, the inclusion of representative quotes ensures that findings are grounded in participants’ own narratives, thereby enhancing confirmability. The final step of the process was the table design and the writing of the research report.

## Results

The results are presented according to the three main themes identified in the data analysis section. First, a characterization of the main consequences of cancer perceived by participants in terms of their body image is presented. This is followed by the main coping strategies used by the participants. Finally, the consequences of the PE program on the participants’ body image are described. Table 1 summarizes this structure of identified themes and sub-themes.

**Table 1:** Themes and sub/themes derived from the data analysis

Cancer consequences	Coping	Physical exercise consequences
- Autoperception	- Discretion	- Physical benefits
- Others’ perceptions	- Empowerment	- Psychological benefits

### Cancer consequences

Changes in the perception of one’s own body image were shown to be a central element in the experience of women diagnosed with breast cancer. These body changes at the time of diagnosis and at the beginning of treatment were reported to be very evident on a visual level, and, in addition, they occurred very soon. Beyond the emotional complexity derived from cancer diagnosis, body transformations are a process that could be overwhelming for many participants:

“Managing the image that you see in the mirror is a complicated process [...] At night I looked in the mirror and saw that I was changing. My hair, my eyebrows, my eyelashes were falling out. And you get fat. That change is… it is brutal.”

However, the awareness of the experienced body changes, as well as the discomfort that comes with it, occurred once the disease stages that supposed a greater risk to patient’s life were overcome:

“This brutal image change has been very complicated. The moment you are no longer afraid of dying, when you believe that everything is starting to go well, that is when you look in the mirror and realize that you no longer recognize yourself.”

An important aspect related to body image had was the perception of other people. In this sense, participants’ concerns about being stigmatized and labeled as “cancer patients” was a central element in terms of discomfort derived from other people’s perception. Likewise, participants showed a deep concern regarding the possibility of being rejected by significant people in their lives, such as children or partners:

“I was more afraid of what my husband would think when he saw me naked than anything else. I was afraid that seeing me like that would cause him rejection.”

The shame and anguish expressed by participants was also related to the fact that they felt their bodies were moving away from the canons of beauty socially legitimized for Western women. The comparison of their bodies with other bodies considered “normative” intensified the feeling of alienation with respect to their own image:

“I have practiced swimming all my life. And after the surgery, I could not enter the locker room without a breast, with all the scars... I entered the municipal pool, and the locker room was full of young girls. How do I take off my swimsuit now, in front of those magazine bodies? I ran to the bathroom, I hid... And I thought ’I cannot do anything comfortably, it is super uncomfortable anywhere.”

### Coping

Practices aimed at discretion, at hiding one’s own body, were used by some participants to confront the development of a negative body image. Those who opted for this strategy remained more at home, wore looser clothing than usual, and/or avoided certain public spaces in order to conceal their body shape or avoid exposure to another people’s gaze:

“I used to wear a lot of tank tops, but now... there are clothes that I just throw away. I did not even want to see them in the closet. I have gained five kilograms; my pants are going to explode. When it comes to outfit, my mind directly leads me to wear skirts, or dresses... things that are not tight, because otherwise I feel very uncomfortable.”

One participant justified this type of actions as:

“it is not just because of me, although I’m also embarrassed to see myself. But it is also because of the sensitivity of others, I feel bad… I do not want them to see me like that.”

This experience reveals, as mentioned above, a duality in the negative perception of body image. On the one hand, the desire to hide one’s own corporeality originated, in an internal dimension of personal rejection, which could be interpreted as a form of symbolic violence in the form of guilt for not having a body aligned with the dominant aesthetic canons. On the other hand, the strategy of discretion also stemmed from a concern for possible perceptions and/or reactions of third parties when faced with the sight of a body visibly affected by the disease.

Contrary to discretion, the empowerment of and among women with breast cancer was shown to be an important narrative of resistance against the restrictive view of beauty in female bodies and, at the same time, of active acceptance of the body changes derived from the cancer disease. The empowered participants thought that the disease experience should not be limited to a passive acceptance of body changes. On the contrary, it meant a stimulus for the vindication and legitimation of his new corporality, and the reconstruction of the dominant aesthetic canons:

“I went to the pool without a prosthesis. If they want to look, let them look.”

The empowered participants not only exposed their bodies in public, but also understood their attitude as a political responsibility aimed at social acceptance of their corporality and challenging the stigmas associated with the disease:

“Changes... are phases that we have to go through. Many physical consequences that we have will remain with us, but here we are, alive. We have to be thankful for that. And I would say we should not hide. If we are not able to normalize our body, we cannot expect others to see it as normal.”

#### Physical exercise consequences

Physical changes as a consequence of PE were a key element in the well-being feeling. Although the majority of women did not directly express a change in their perception body image, they did express noticing obvious changes in their physical appearance (e.g., increase in muscle mass legs and arms, improvement in arm mobility, decreased body weight, improved posture) and some patients even reported a perceived improvement in their physical self-image:

“I gained 20 kg and had muscle atrophy. Now I don’t feel it that bad, in terms of my body image, at all. I mean, I still need to lose a few kilos to feel completely well, but it is a process that I feel is going very well. I look much better on a physical level.”

Conversely, while physical activity was beneficial in attenuating treatment-induced physical alterations, in certain cases, the improvements achieved through the PE program were deemed insufficient:

“Exercise has helped me to improve my muscle tone and strength and to lose weight, […]. And I feel very good doing exercise. But I still feel very deteriorated in my physique, and that it is because of all the adverse effects that I have had.”

On the other hand, participants showed greater body confidence since their started into the PE program. They evaluated their appearance more positively, which contributed to having positive thoughts and feelings in relation to their body, and increase their levels of self-esteem. One participant verbalized it like this:

“The flaccidity that you see, the loins… I look much better now with exercise. When I see myself in the mirror it is totally different, I have more confidence.”

Of particular relevance is the fact that the reported changes were not limited to the appreciation of physical appearance or capabilities, but also extended to perceptions of body functionality. Several participants reported experiencing moments during the PE sessions in which they temporarily forgot about their illness. Although pain and cancer-related fatigue were sometimes cited as barriers to engaging in PE, participants also described increased overall well-being, a reduction in treatment-related side effects, and enhanced body acceptance, self-respect, and a more favorable body image:

“When I come here, I feel great. As if I were 20 years old, as if I weren’t sick, and as if I were capable of anything the physical trainer asks me to do.”

In this context, the PE program appears to support the development of a more positive body image, fostering greater acceptance and appreciation of the body, which in turn contributes to improved interpersonal relationships:

“Exercise makes me feel more optimistic, more active… and that translates into better social relationships.”

Psychological changes resulting from the PE program also showed to be a key element in the feeling of well-being. All participants emphasized how PE facilitated a greater appreciation of their bodies, enhanced their self-esteem, and fostered positive perceptions of their bodies. In this regard, they stated that engaging in a PE group program was an important factor to feel motivated, socialize and empathize with women facing similar challenges. Moreover, the opportunity to openly share both physical and emotional symptoms related to their illness provided a sense of companionship, understanding, and empowerment in their fight against cancer:

“This group is very important for me, because aside from exercise, it has helped me to feel more relaxed since I have colleagues who are further along in the disease and can share with me important experiences that I am about to face. In this sense, you feel calmer because you know more or less what is going to happen, what to expect at each moment. They also offer advice, such as, ‘This is good for your eyelashes.’”

## Discussion

Female breast cancer patients have to cope with traumatic physical and psychological experiences caused by the diagnosis and treatment that affect patients’ daily life, social activities, relationships, quality of life and also associated with body image changes [20]. Considering that body image is a multidimensional construct that encompasses perceptions, thoughts, feelings and behaviors, in relation to the appearance, functions and capabilities of the body [9], up to 77% of breast cancer patients experience some degree of discomfort with their body image [21]. PE has been identified as an effective strategy to mitigate these experiences [12]. Therefore, the purpose of the present study was to know the body image perceptions of female breast cancer patients who took part in a PE program.

Among the results, the emotional impact derived from the physical changes that occur after the diagnosis should be highlighted, especially as an effect of treatments. A significant concern of the participants was associated to the perception of their relatives, such as their partners, who could negatively react to the body changes resulting from cancer. The rejection of these “sick bodies” has been documented in previous studies [22, 23]. However, focus group conversations revealed that this fear was more of a belief than a reality. Thus, part of this sensation can be explained by an element of self-rejection driven by the internalization of beauty ideals, from which the participants’ bodies move away once they begin to change as a consequence of their illness [8].

The acceptance of a new corporality after breast cancer diagnosis is not a uniform process, but rather a gradual and variable one [23]. Faced with these body changes, the participants deployed two types of coping strategies, very different from each other: discretion and empowerment. Empowerment stood out as a significant approach for many participants, especially among those who were older in terms of both, age and time living with the diagnosis, while recently diagnosed women were more likely to adopt strategies of discretion (research such as Cash and Smolak [9] suggests that life stage is important in body image perception, and it is more negative in young breast cancer patients). Active acceptance of body changes was shown to be an important element for the adaptation process, in which social support played a crucial role. In this sense, training partners were the most important source of support. This is consistent with previous studies showing how, in the context of breast cancer patients, an exercise group is an important factor in promoting peer social support [12, 24]. This effort to reconstruct the dominant beauty canons by accepting the post-diagnosis and/or post-treatment body, together with emotional support, represent psychosocial factors that can contribute to a more positive body image [25].

The results show from a qualitative point of view, how a structured PE program can improve physiological and psychological function, which can in turn, promote physical and emotional well-being [12, 26]. Beyond the physical changes described by participants (e.g., weight loss or muscle mass increase), exercise also played a crucial role in fostering a positive relationship with their bodies in terms of perception, appreciation, attitude, affectivity and behavior. According to the literature, body image is linked to health condition and physical functioning [27] what suggests that changes in physical appearance identified by participants in the present study also contributed to improve their body image [28]. Furthermore, some of the participants revealed higher levels of self-efficacy caused by PE, and it is noteworthy that this self-efficacy has been previously associated with better self-esteem and body image perception [29].

The results of the present study provide new knowledge related to body image in the context of breast cancer, emphasizing that the inclusion in a PE program can have a positive impact on body perception among female breast cancer patients. Healthcare professionals can use this information to better understand the impact of PE on breast cancer patients and their experiences, highlighting the importance of including PE as an essential component of rehabilitation programs during adjuvant treatment or after the oncology treatment. It is important to emphasize that qualitative studies play a fundamental role in research like ours, as they allow patients to articulate their perceptions and experiences in a more open and nuanced manner, providing a richer and deeper understanding of the subjective impact that a physical exercise program can have on self-perceived body image. This approach is essential to capture the individual variations in women’s experiences, as each experience is unique and may be influenced by contextual, emotional, and social factors.

This study has two main limitations that should be acknowledged. Firstly, the cross-sectional design provides only a snapshot of participants’ perceptions, which may be affected by recall bias, particularly among individuals who have lived with breast cancer for a long time. Therefore, it is important to continue exploring the impact of breast cancer on women’s body image longitudinally, adopting an approach that considers this experience to be dynamic and multifaceted, which requires data to be collected at multiple time points. Secondly, breast cancer often involves aggressive treatments that can lead to visible physical changes, such as hair loss and nail damage, which may be difficult for physical exercise programs to address. In this regard, future studies may benefit from analyzing the experiences of participants who have undergone such changes separately, to better understand the extent to which physical exercise can contribute to improving body image in these specific circumstances.

## Conclusion

Breast cancer and its treatments induce significant physical changes. In many cases, the societal pressure to conform to dominant aesthetic standards can contribute to the development of a negative body image, even in the presence of PE within structured programs. However, the PE program examined in this study primarily contributed to an enhanced positive body image, particularly in areas related to physical appearance, emotional well-being, and cognitive function. PE is crucial as an adjunctive treatment during and after breast cancer therapy, and thus should be integrated into cancer rehabilitation programs (both pre- and post-surgery). Furthermore, interdisciplinary collaboration among healthcare professionals, including those in medicine, nursing, physiotherapy, and sports sciences, is essential to ensure the success of these interventions.

## Data Availability

No datasets were generated or analysed during the current study.

## Appendix

Interview Guide: Focus Group Questions.

1. What does body image mean to you?
2. How do you feel about your body image after being diagnosed with breast cancer?
3. How has breast cancer affected your body image?
4. What role does physical exercise play in your perception of body image?
5. What benefits have you experienced in terms of body image from participating in the exercise program?
6. What challenges or barriers have you faced when trying to follow an exercise program after having breast cancer?
7. How has your body image changed since you began participating in the exercise program?
8. How do you feel about your body overall after having breast cancer and participating in a physical exercise program?
9. How have breast cancer and physical activity affected your relationship with your body and your overall health?
10. What recommendations would you give to other women with breast cancer who are considering joining a physical activity program?
